# Engagement With a Digital Platform for Multimodal Cognitive Assessment and Multidomain Intervention in a Japanese Population: Pilot, Quasi-Experimental, Longitudinal Study

**DOI:** 10.2196/15733

**Published:** 2019-10-25

**Authors:** Jordan Glenn, Erica Nicole Madero, Michelle Gray, Nami Fuseya, Mari Ikeda, Tomoo Kawamura, Yoshiko Arita, Nick Thomas Bott

**Affiliations:** 1 Neurotrack Technologies Inc Redwood City, CA United States; 2 Exercise Science Research Center Department of Health, Human Performance and Recreation University of Arkansas Fayetteville, AR United States; 3 Nipponkoa Himawari Life Insurance, Inc Tokyo Japan; 4 Sompo Holdings Tokyo Japan; 5 Department of Medicine School of Medicine Stanford University Palo Alto, CA United States

**Keywords:** cognitive decline, Alzheimer disease, lifestyle risk reduction, digital health, FINGER, neurocognitive tests, cognition, dementia

## Abstract

**Background:**

As the global prevalence of dementia continues to rise, multidomain lifestyle interventions that address modifiable risk factors associated with pathological cognitive decline are increasing. Although some digital options have been developed to increase the reach and scalability of these programs, because of cultural differences, the efficacy of the programs in one population cannot easily be generalized to populations in other countries.

**Objective:**

This investigation aimed to examine the usability and engagement of a digitally delivered multidomain cognitive lifestyle intervention developed in the United States for a Japanese population.

**Methods:**

This feasibility investigation utilized a quasi-experimental, single-arm, nonrandomized, longitudinal design where participants engaged in the behavioral intervention on a smartphone. Of the 559 participants that initially enrolled (age: mean 51 years, SD 7.5 years; 51.7% female [289/559]), 242 completed the final testing trial. Participants enrolled in a multidomain lifestyle program that consisted of (1) psychoeducational material, (2) physical activity tracker, (3) nutrition tracker, (4) audio-based meditations, and (5) health coaching. Engagement with the program was assessed through the total number of app sessions and the use of the exercise, diet, and meditation tracking features within the app. The total number of minutes exercised was collected through subjective user inputs, and nutrition was quantified by the Mediterranean-DASH Intervention for Neurodegenerative Delay diet adherence score.

**Results:**

Significant relationships existed between overall nutrition score and frequency of nutrition tracking (*r*=0.18), frequency of physical activity tracking (*r*=0.19), and the total number of minutes exercised (*r*=0.22). Total minutes exercised was significantly correlated with total app sessions (*r*=0.57), frequency of physical activity tracking (*r*=0.85), frequency of nutrition tracking (*r*=0.64), number of times participants meditated (*r*=0.46), and total lessons read (*r*=0.36). The number of completed lessons was significantly related to frequency of physical activity tracking (*r*=0.40), frequency of nutrition tracking (*r*=0.43), the total number of times participants meditated (*r*=0.35), and total minutes exercised (*r*=0.33). Dividing the cohort into two groups based on lesson completion (<10 lessons completed vs ≥10 lessons completed), significant differences were observed between the total minutes exercised, frequency of physical activity tracking, frequency of nutrition tracking, and total number of times participants meditated (all *P* values <.01).

**Conclusions:**

Overall, this cross-cultural feasibility study in Japanese users demonstrated that the various engagement metrics were significantly correlated, and greater engagement was related to improved nutrition scores and increased time exercising. In addition, the relationships between lesson completion and other engagement metrics suggest that there may be value in exploring mechanisms that enhance lesson completion. Future research should examine the program in randomized control trials to more rigorously evaluate program efficacy.

## Introduction

### Background

Characterized by declines in mental ability severe enough to interfere with daily life, Alzheimer disease (AD) poses a serious worldwide challenge as it relates to patients, their caregivers, and health care systems. Projections indicate that the global prevalence of AD is expected to triple to over 150 million individuals between 2015 and 2050 [1]. In the United States alone, AD costs are projected to grow from US $290 billion in 2019 to US $1.1 trillion in 2050, representing a 400% increase, whereas AD diagnoses are projected to increase by approximately 150%, from 5.5 million to 13.8 million over that same time span [2].

Although there is a massive cost associated with the disease once diagnosed, AD tends to go undetected for long periods because of a prolonged preclinical phase [3,4]. In this phase, neuronal and neurobiological changes can occur for years or decades before noticeable symptoms appear. Before the development of clinically detectable cognitive issues, people commonly experience a phenomenon termed as subjective cognitive decline (SCD) [5]. SCD is defined as subjective changes in memory and cognition that are perceived by the individual but are not associated with clinically measurable abnormalities [3,6]. Individuals experiencing SCD are considered at risk for developing dementia, specifically AD [7-9]. If left unchecked, SCD can evolve into mild cognitive impairment (MCI), an intermediate between normal cognitive function and diagnosable AD [6,7].

Age is the number one risk factor for AD, and the probability of being diagnosed with AD nearly doubles every 5 years after the age of 65 years [8]. Among adults older than 65 years, approximately 1 in 5 currently suffer from AD [9]. This number has increased dramatically in the United States since 2000, with an increase in diagnoses of 145% [9]. Although these numbers are alarming, they are dwarfed by the massive growth in Japan’s aging population. Compared with the rest of the world, Japan has the largest aging population, with 33% of the population aged 60 years or older [10,11]. Furthermore, Japan’s older population is projected to continue growing through 2050, reaching an unprecedented 42% of the population. This is critically important as the number of Japanese adults with dementia is estimated to be approximately 4.6 million, comprising nearly 15% of the older adult population [12]. When individuals with MCI are included, this number rises to approximately 8.6 million, constituting 30% of Japanese older adults [12]. The estimated cost of dementia in Japan in 2014, defined as the sum of costs for health care and formal and informal care, was approximately ¥14.5 trillion, or an estimated 3% of the nation’s gross domestic product [13].

Through accumulated and ongoing randomized control trials, it is increasingly accepted that the development of AD is multifactorial, with a combination of modifiable and unmodifiable risk factors ultimately leading to the onset and progression of the disease [14-16]. As a result, it is difficult to conceptualize risk reduction for AD as a single-factor solution; rather, it is recommended to focus on multiple behavioral risk factors associated with disease development and progression [17]. The primary modifiable risk factors associated with AD are physical activity, diet, sleep, stress, social interaction, and cognitive engagement. Improvement in any of these factors may reduce the overall risk for disease onset and progression [16]. Furthermore, the ability to improve these risk factors at an individual level can have an overall beneficial effect on cognitive aging trajectories.

The nearly ubiquitous penetration of the wireless internet and subsequent adoption of smartphone technology offer an opportunity to address the demands and burdens associated with pathological cognitive decline by improving accessibility to digital solutions [18] and ultimately reducing associated health care costs [19]. To date, a number of multidomain interventions have focused on behavioral risk mitigation and enhancement of cognitive performance. These include the Finnish Geriatric Intervention Study to Prevent Cognitive Impairment and Disability (FINGER) in Finland [16], US Study to Protect Brain Health Through Lifestyle Intervention to Reduce Risk in the United States [20], Singapore intervention study to prevent cognitive impairment and disability in Singapore [21], Maintain Your Brain in Australia [22], Multidomain Alzheimer's Prevention Trial (MAPT) in France [23], Prevention of Dementia by Intensive Vascular Care in the Netherlands [24], and all other studies under the scope of World Wide Fingers [25]. Although all these interventions contain Web-based components, none of them are completely digital, with the exception of Australia’s Maintain Your Brain [22], thereby limiting the scalability and scope of the intervention’s effectiveness. In fact, the only fully digital interventions focused on improving the aforementioned modifiable risk factors for pathological cognitive decline have been the Body Brain Life [26] and the Virtual Cognitive Health (VC Health) study [27,28], wherein users could access the entirety of the program via the internet through a laptop or desktop computer.

### Objectives

On the basis of the results of the VC Health study, we developed the Neurotrack Memory Health Program (MHP) that transitions the form factor from a Web-based intervention to a smartphone. Combining aspects of physical activity, diet, sleep, stress, social interaction, and cognitive engagement, this new method of program delivery expands the scope and accessibility of the behavioral intervention, which are limiting factors in receiving proper care [18]. Given the novelty of the program and method of delivery, our primary aim in this initial investigation was to evaluate the program’s feasibility and acceptability of use in free-living individuals. On the basis of the current scope of the Japanese aging population and the disease-associated health care costs [12,13], this study investigated the feasibility and acceptability of the Neurotrack MHP in the Japanese population.

## Methods

### Study Overview

This feasibility investigation utilized a quasi-experimental, single-arm, nonrandomized, longitudinal (16-week) design in which participants used the Neurotrack MHP smartphone app to engage in a behavioral intervention designed to improve risk factors related to cognitive decline. As this program was originally an English product, there were requirements to make it accessible to Japanese populations, including translation to Japanese and adjustment of certain recommendations to be more culturally applicable (see below for a detailed description of the localization process). The smartphone app was used to collect all study data and monitor completion of program-related tasks. Participants completed all study procedures remotely via their smart device. The research protocol was approved for retrospective exemption by the institutional review board at the University of Arkansas for the analysis of deidentified data.

### Participants

Participants in this investigation included employees and parents of employees of a large Japanese insurance company (Sompo Holdings, Tokyo, Japan); the program was offered as a free opportunity for all participants. It was important that this program was offered free of charge to remove incentivization bias on behalf of the participants. Participants were recruited through email outreach, flyers, and word of mouth throughout the company. As this was offered as an employee and relative benefit, the only requirements were that participants speak Japanese (to understand the program), be older than 40 years, and have access to a smartphone with internet access; no one was excluded from participation based on location or current health status. The rationale for including participants aged 40 years and older is based on epidemiological evidence suggesting that this is the age at which AD pathology begins to accumulate [29,30]. In addition, engaging in healthy habits in midlife may extend life spans by as many as 14 years in women and 12.2 years in men [31], and among middle-aged adults, 11.2% report SCD [32].

### Localization for Japan

Once the English version of the program was finalized, an initial translation was provided by a bilingual (English and Japanese) speaker native to the Japanese language and culture. This was a requirement to ensure the nuances of the educational components were not lost in the translation process. After the program was translated, it was reviewed by another bilingual (English and Japanese) speaker native to the Japanese language and culture. After consensus was reached between each of the bilingual speakers regarding content, it was piloted with 5 individuals living in Japan who met the criteria for program delivery. Japanese individuals used for the pilot trial did not speak English to ensure a clear understanding of the content. After pilot data were collected, adjustments were made incorporating participant feedback. This process resulted in the creation of a fully Japanese version of the original English program.

### Study Intervention

Once recruited, participants were sent an email to join the program, received an invitation to download the Neurotrack MHP app to their personal device, and were instructed to set up their in-app profile. To replicate the fully digital approach utilized in the VC Health study, participants were never required to report to a testing center or laboratory. After profile setup, participants completed a baseline survey to assess initial health status and behaviors (Table 1).

The survey questions were selected to understand the baseline risk factors commonly associated with AD (physical activity [33], diet [34], sleep [35], depression [36], and anxiety [37]).

**Figure 1:**
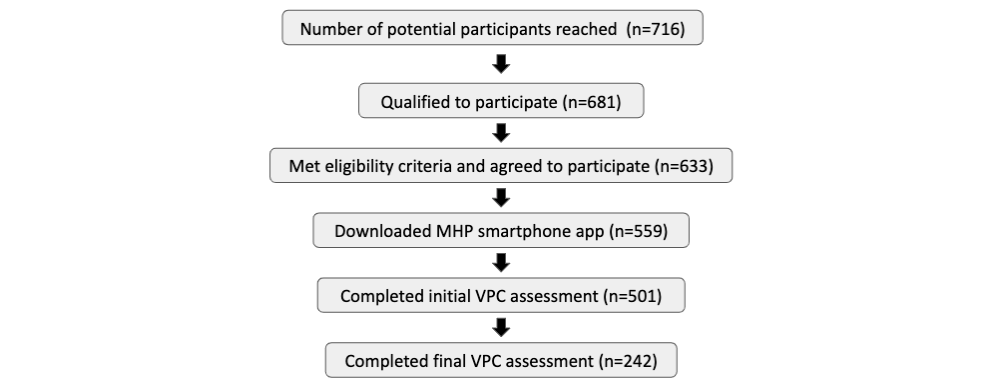
Participant study funnel. MHP: Memory Health Program; VPC: visual paired comparison.

Participants were then required to complete a visual paired comparison (VPC) eye-tracking assessment delivered through the Neurotrack MHP app [38,39]; a detailed description of the VPC task is included below. All other components of the program were blocked until after completion of the initial VPC test, which provided a baseline memory performance score (Figure 1).

**Figure 2:**
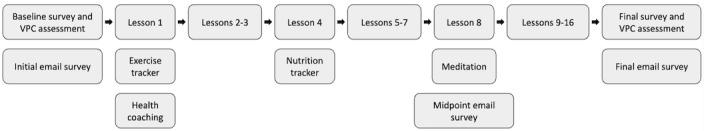
Timeline of the Memory Health Program and study protocol. VPC: visual paired comparison.

After completion of the initial VPC assessment, participants had full access to the Neurotrack MHP. The Neurotrack MHP offered a number of primary functions that allowed users to track and engage in behaviors that may reduce the risk of cognitive decline. These features included (1) psychoeducational material, (2) self-reported physical activity tracker, (3) self-reported nutrition tracker, (4) audio-based meditations, and (5) health coaching. Each feature is described in detail below. These features were released on a timed schedule in an effort to gate the amount of information a participant was presented at any one time (Figure 2). Participants were encouraged to utilize the app daily to track their health behaviors and engage with the associated features. At the end of the 16-week intervention, all participants were provided access to a follow-up VPC assessment that could be taken again through the smartphone app.

**Table 1:** Baseline survey questionnaire provided to all participants. Note: the Patient Health Questionnaire-4 was used to assess depression and anxiety.

Question	Response type
On average, how many minutes of physical activity do you complete per week?	Numeric
During a normal week, how many days do you eat fish?	Numeric
During a normal week, how many days do you eat vegetables?	Numeric
During a normal week, how many days do you sleep more than 7 hours a night?	Numeric
Over the last 2 weeks, how often have you been bothered by the following problem: Feeling nervous anxious or on edge	Categorical (PHQ-4^a^)
Over the last 2 weeks, how often have you been bothered by the following problem: Feeling down, depressed or hopeless	Categorical (PHQ-4)
Over the last 2 weeks, how often have you been bothered by the following problem: Not being able to stop or control worrying	Categorical (PHQ-4)
Over the last 2 weeks, how often have you been bothered by the following problem: Little interest or pleasure in doing things	Categorical (PHQ-4)

^a^PHQ-4: Patient Health Questionnaire-4.

### Intervention Components and App Functionality

#### Visual Paired Comparison Task

The VPC task was completed on the participant’s device through the embedded Web camera. The construction of the VPC task has been explained in detail elsewhere [27,38,39]. Briefly, VPC tasks utilize eye-tracking technology to assess visual recognition memory by quantifying the time a participant spends viewing novel images as opposed to previously viewed images [39-41]. The VPC is administered in 2 separate phases: a familiarization phase and a testing phase.

During the assessment’s familiarization phase, participants were presented with pairs of identical visual stimuli, each for 5 seconds. During the test phase, subjects were presented with pairs of visual stimuli (images) in the same format as the familiarization phase; however, this phase includes only 1 image from the familiarization phase and 1 novel image. The proportion of time a participant spent gazing at the novel image relative to the total viewing time produced a novelty preference score, with higher scores representing better declarative memory performance and lower scores indicating worse declarative memory performance [38,39]. Eye movements were tracked and scored. Detailed scoring information is published elsewhere [38].

#### Program Features and Functions

The psychoeducational material was organized into a series of 17 individual lessons (1 introductory lesson + 16 weekly informational lessons) that were presented to participants weekly. The content included information on the aforementioned lifestyle behaviors related to cognitive decline and general behavior change concepts such as goal setting and problem solving. Participants were asked to read the new lesson each week but were allowed to revisit the previous lessons at any point throughout the program. The physical activity tracker allowed participants to record their daily activities and review them in the app. The nutrition tracker was developed based on the Mediterranean-DASH Intervention for Neurodegenerative Delay (MIND) diet [42-44]. Participants were asked to track the foods they consumed throughout the day based on the categorical breakdown of the foods in the dietary pattern [45].

The meditation feature included categories related to mindfulness, sleep, and stress. All 3 categories had 2-, 5-, and 10-min options for the participant to choose based on their preferences and needs. For social engagement, there were dedicated lessons with suggestions on how to become more engaged within the community. Finally, participants had access to a personal health coach. The health coach was available for participants to ask questions related to cognition, cognitive decline, or behavioral health. For the purposes of this intervention, the coaches had backgrounds in personal training, nutrition, nursing, and social work.

### Outcome Measures

#### Primary Outcome

Program engagement was the primary outcome of this pilot study. Participant engagement was evaluated based on the total sessions within the app; number of lessons read; and utilization of physical activity, nutrition, and meditation features.

#### Secondary Outcomes

Self-reported health was assessed through behavioral data collected in the app. These behaviors included anxiety and depression (Patient Health Questionnaire-4 [PHQ-4]), minutes of physical activity completed, nutrition scores, and sleep quantity.

### Data Analysis

R 3.5.2 [46] was used to conduct all analyses. Descriptive statistics were calculated for engagement and variables related to self-reported health. *t* tests were conducted to evaluate sex differences. Engagement variables included the number of lessons completed (ie, total number of times lessons were viewed), number of times physical activity was tracked, number of times nutrition was tracked, number of times meditation was tracked, and number of times VPC test results were checked. In addition, variables related to self-reported health were defined as anxiety, depression, physical activity, nutrition, and sleep quantity. Paired *t* tests were utilized to determine differences between pre- and postscores for variables related to self-reported health status.

For relationship analyses, Pearson product-moment correlation coefficients were utilized to investigate the relationships between individual engagement variables as well as the relationships between these in-app engagement variables and lessons completed. Similar relational analyses were conducted comparing overall nutritional scores and total minutes exercised with engagement variables.

In addition, change scores were calculated for self-reported health status and related behavior variables. For these change scores, Parson product-moment correlation coefficients were also utilized to investigate relationships between baseline scores and score changes over the course of the program.

Finally, the relationships between the number of distinct lessons completed and other engagement data were evaluated through Pearson product-moment correlation coefficients. As mentioned previously, although the participants could view the lessons as many times as they wanted, there were 17 distinct lessons released throughout the course of the program (1 introductory lesson + 16 weekly informational lessons). On the basis of the observed relationships, participants were separated into groups based on the number of distinct lessons completed (n<10 and n≥10). Paired *t* tests were used to determine the differences in engagement and self-reported health based on cutoffs for completion of distinct lessons.

## Results

The participant enrollment funnel is presented in Figure 2; of the initial 716 individuals targeted to be a part of the pilot program, 559 downloaded the app and were classified as enrolled. Of those enrolled, 501/559 (89.6%) completed the initial VPC and 242/559 (43.3%) completed the final VPC. Of those completing the final VPC, 51.7% were female and 49.3% were male.

No sex differences were observed for age (females: mean 51 years, SD 7.9 years; males: mean 51 years, SD 7.1 years; *P*=.97), total number of sessions (females: mean 92, SD 79; males: mean 81, SD 69; *P*=.26), frequency of physical activity tracking (females: mean 29, SD 34; males: mean 23, SD 29; *P*=.18), total lessons read (females: mean 22, SD 13; males: mean 19, SD 10; *P*=.13), total results checked (females: mean 5, SD 4; males: mean 5, SD 5; *P*=.99), or distinct lessons loaded (females: mean 13, SD 5; males: mean 12, SD 5; *P*=.38). Significant differences were observed between sexes for frequency of nutrition tracking (females: mean 36, SD 48; males: mean 22, SD 34; *P*=.002) and frequency of meditation tracking (females: mean 7, SD 10; males: mean 4, SD 5; *P*=.008). Differences were also observed in baseline VPC scores (females: mean 86.8, SD 9.2; males: mean 84.1, SD 7.3; *P*=.02); however, there was no significant difference in VPC change scores between males and females. In addition, the total number of minutes exercised were not significantly different (females: mean 1007, SD 1316; males: mean 856, SD 1083; *P*=.32) between males and females, but there were significant differences (females: mean 7.9, SD 1.7; males: mean 7.2, SD 1.5; *P*=.03) in overall nutritional score.

Pre- and postscores for self-reported health variables are reported in Table 2. Significant differences between timepoints were observed for the number of days per week eating fish (*P*=.01); however, no other significant changes were observed. In addition, baseline VPC scores were not significantly different from final scores, indicating no cognitive changes occurred throughout the intervention.

**Figure 3:**
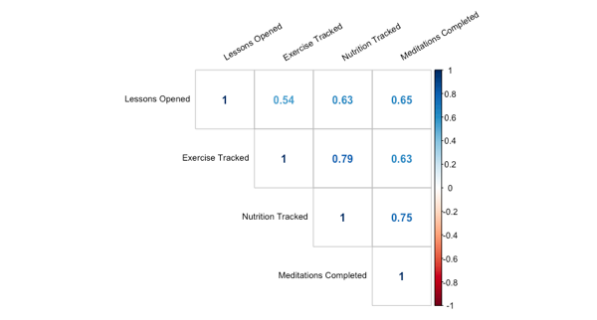
Relationships between engagement variables associated with the Memory Health Program.

As defined previously, the engagement variables assessed for this investigation included lessons completed, frequency of physical activity tracking, frequency of nutrition tracking, frequency of meditation tracking, and frequency of results viewing. Number of lessons completed was significantly correlated with the frequency of physical activity tracking, frequency of nutrition tracking, and frequency of meditation tracking. See Figure 3 for a visual representation of individual relationships and correlation coefficients (please note that all relationships in Figure 3 are significant).

**Table 2:** Analysis of pre- and postscores for Memory Health Program data.

Variable measured	Prescore, mean (SD)	Postscore, mean (SD)	*P* value
Visual paired comparison score	84.1 (8.1)	84.9 (7.2)	.09
Days per week eating fish	2.3 (1.2)	2.6 (1.2)	.01
Days per week eating vegetables	6.0 (1.5)	6.0 (1.5)	.60
Days per week sleeping more than 7 hours each night	2.8 (2.1)	3.0 (2.1)	.10
Total score (Patient Health Questionnaire-4)	2.4 (2.2)	2.6 (2.6)	.12

**Figure 4:**
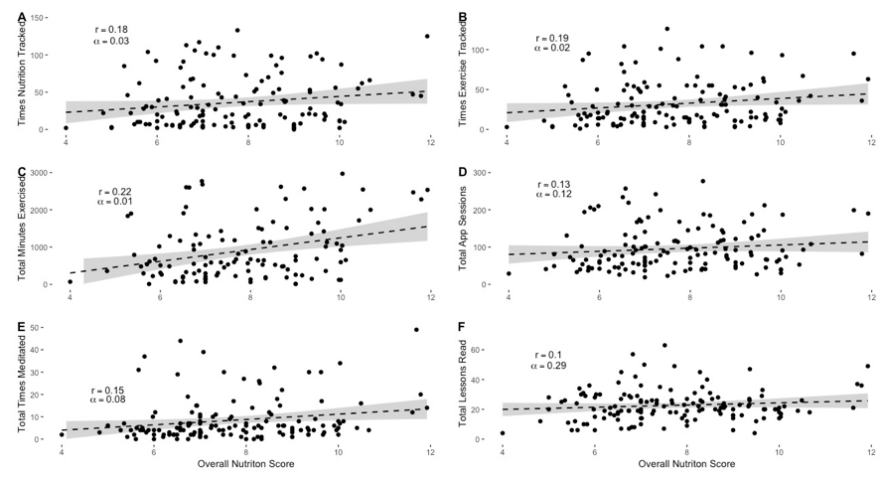
Relationship of overall nutrition score with other engagement variables.

When evaluating overall nutrition score, a mean final score of 7.6 (SD 1.7) was obtained by the participants (calculated based on the MIND diet recommendations [34]). Significant relationships existed between overall nutrition score (Figure 4) and frequency of nutrition tracking (*P*=.03; *r*=0.18), frequency of physical activity tracking (*P*=.02; *r*=0.19), and the total number of minutes exercised (*P*=.01; *r*=0.22). Nonsignificant correlations were observed between overall nutrition score and the total number of app sessions (*P*=.12, *r*=0.13), number of times participants meditated (*P*=.08; *r*=0.15), or total lessons read (*P*=.29; *r*=0.10). These relationships between engagement and dietary outcomes are important as scoring a 7.5 to 9.5 on the MIND diet has been associated with a 35% decreased risk of disease development (for a detailed description of the scoring structure, please see the study by Morris et al [34]).

**Figure 5:**
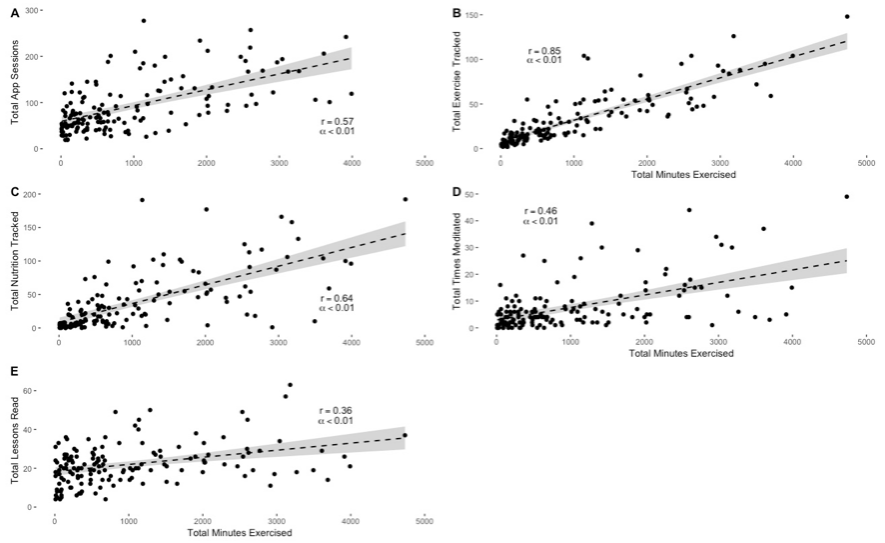
Total minutes exercised with other engagement variables.

Total minutes exercised over the course of the intervention were 936 (SD 1211) per participant. Total minutes exercised (Figure 5) was significantly correlated with total app sessions (*P*=.002; *r*=0.57), frequency of physical activity tracking (*P*<.001; *r*=0.85), frequency of nutrition tracking (*P*=.004; *r*=0.64), number of times participants meditated (*P*=.008; *r*=0.46), and total lessons read (*P*=.007; *r*=0.36). Physical activity plays a significant role in promoting cardiovascular health and reducing risk of cognitive decline [47,48]. These relationships suggest that increased Neurotrack MHP engagement supports increases in total minutes of physical activity, potentially leading to future positive health outcomes.

Change scores were calculated for self-reported health behaviors by subtracting the prescore from the postscore. Significant negative relationships existed between self-reported baseline score and changes throughout the program. Specifically, these relationships were observed for minutes of activity per week (*P*=.003; *r*=−0.45), number of days eating fish (*P*=.007; *r*=−0.30), and days eating vegetables (*P*=.004; *r*=−0.45). These data indicate that individuals initially reporting lower levels of the behavior improved, whereas those initially reporting higher levels of the behavior remained stable.

**Figure 6:**
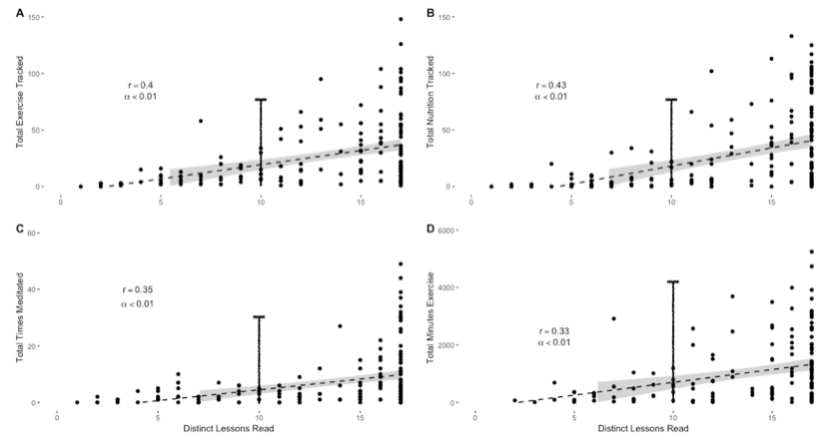
Relationships between the total number of distinct lessons completed and other engagement variables. Note: drop lines indicate the 10-lesson cutoff.

The total number of distinct lessons completed (out of the 17 provided) were significantly related to the engagement variables (Figure 6). Specifically, distinct lessons completed were significantly related to the frequency of physical activity tracking (*P*=.004; *r*=0.40), frequency of nutrition tracking (*P*=.004; *r*=0.43), total number of times participants meditated (*P*<.007; *r*=0.35), and total minutes exercised (*P*=.008; *r*=0.33). On the basis of these relationships, a cutoff of 10 distinct lessons completed was determined to be an inflection point for engagement. Paired *t* tests were used to determine the differences in engagement and self-reported health based on a 10-lesson cutoff point.

As shown in Table 3, no differences were observed in VPC scores (*P*=.55) between lesson completion groups (<10 lessons vs ≥10 lessons); however, significant differences were observed for total minutes exercised, frequency of physical activity tracking, frequency of nutrition tracking, and total number of times participants meditated (all *P* values <.001). When comparing change scores between lesson completion groups for self-reported health, there were no differences observed between groups for PHQ-4 score (*P*=.48), minutes of activity completed per week (*P*=.59), days per week eating fish (*P*=.93), days per week eating vegetables (*P*=.46), or nights per week sleeping more than 7 hours (*P*=.79).

**Table 3:** Participant engagement data based on completion of 10 or more distinct lessons.

Engagement variable	<10 distinct lessons completed, mean (SD)	≥10 distinct lessons completed, mean (SD)	*P* value
Total minutes exercised	378 (582)	1181 (1131)	*<*.001
Total number of times physical activity was tracked	8.9 (10.0)	34.1 (32.3)	*<*.001
Total number of times nutrition was tracked	5.6 (8.7)	41.6 (46.6)	*<*.001
Total number of times participants meditated	2.3 (2.5)	8.9 (12.0)	*<*.001
Visual paired comparison score	−1.5 (5.6)	−0.9 (7.2)	.55

## Discussion

### Principal Findings

The primary aim of this pilot investigation was to evaluate the Neurotrack MHP’s feasibility and acceptability of use in free-living Japanese adults. By combining aspects of physical activity, diet, sleep, stress, social interaction, and cognitive engagement, this new method of program delivery aimed to expand the scope and accessibility of the intervention, which are currently limiting factors in receiving proper guidance through behavior change for cognitive health [18]. The choice to utilize a Japanese population was based on the costs related to cognitive decline and disease care [12,13] of their aging population.

This investigation focused on a multidomain approach to cognitive health; multidomain lifestyle interventions are recommended as the best strategy to ameliorate cognitive decline because of the synergistic nature of multiple areas of lifestyle modification. This is similar to the original FINGER study [16] that served as the framework for this investigation. The FINGER study showed a 25% increase in global cognition, with specific increases of 83% and 150% in executive functioning and processing speed, respectively. However, these increases were not able to be mapped to a single behavioral intervention, given the multidimensional nature of the intervention. Furthermore, the initial study evaluating the Web version of this digital intervention was unable to delineate a single factor relating to healthy improvements [28], justifying the need for an intervention that provides a more personalized approach (ie, not all participants will be able to exercise 60 min per day). In this study, we present a similar result wherein significant relationships were observed between the various types of engagement within the program (reading lessons, tracking physical activity, tracking nutrition, and meditating). Of these variables, only lessons followed a strict cadence (1 released per week), thus presenting a potential candidate for other engagement metrics to follow. All engagement metrics were interrelated, and although it is reasonable to infer that reading the weekly lessons may be a strong factor for continued engagement, it is impossible to determine a causal relationship from the current data, and further research is required to evaluate this concept.

Significant relationships were also observed between a number of the behavioral health change scores and baseline scores, indicating more improvements in participants reporting lower initial levels, whereas individuals reporting initial higher levels remained stable. Specifically, these increased change scores were observed in minutes of physical activity completed per week, number of days eating fish, and number of days eating vegetables. Although these changes are not necessarily causal to improvements in cognition, these improvements in healthy behaviors are similar to those reported in the FINGER study [16,49] where “fish intake at least twice/week,” “daily intake of vegetables,” and “physical activity 2 or more times/week” were increased after the 2-year intervention. In this study, these changes were independent of changes in cognition, as assessed by the VPC task; however, given the length of time required to observe improvements in cognition, as observed in previous multidomain behavior change interventions—FINGER (24 months) [16,49], MAPT (36 months) [23], Healthy Aging Through Internet Counseling in the Elderly (18 months) [50], and VC Health (12 months) [27,28]—it is likely that the intervention used in this study was too short to result in a substantial change in cognitive performance. Furthermore, this study did not limit recruitment to *at-risk* participants as was done in previous investigations [16,27,28,49], thus limiting the ability for change given the participants’ baseline cognitive performance. The similar changes in behavioral health factors observed in this study as compared with the FINGER protocol [16] indicate the potential for similar improvement in cognitive performance among at-risk participants following a longer intervention duration. As a result, future investigations will need to further evaluate the long-term efficacy of the Neurotrack MHP on cognitive health outcomes. One such example of this type of investigation is the Digital Cognitive Multidomain Alzheimer’s Risk Velocity [51], which is aiming to determine if this type of a broadly disseminable digital program can slow cognitive decline in at-risk participants to ultimately delay or prevent AD onset.

### Physical Activity Outcomes

Additional behavioral outcomes from this investigation included the number of minutes of activity completed and nutrition scores. As expected, the total number of minutes exercised was significantly related to the frequency of physical activity tracking. The total number of minutes exercised was also related to the total app sessions, frequency of nutrition tracking, number of times participants meditated, and total lessons read. The total minutes exercised averaged almost 1000 per participant over the course of the intervention or approximately 16.5 hours of total physical activity completed over the course of the program. These data, in combination with change scores in self-reported physical activity inversely correlated with baseline levels, indicate that participants increased their activity levels throughout the program, without decreases among individuals reporting initially higher baseline values. Physical activity plays a significant role in promoting cardiovascular health and reducing risk of cognitive decline [47,48], and it has been reported that every additional hour of light-intensity physical activity is significantly associated with higher brain volumes (even among individuals not meeting current recommended guidelines) [52].

However, it should be noted that the participants in this study did not reach activity duration thresholds reported in previous investigations suggested to enhance cognition. For example, the Nurses’ Health Study showed that women who walked 90 min/week demonstrated higher global cognition scores compared with women who walked less than 40 min per week [53]. Furthermore, it has been reported that individuals who are physically active for approximately 30 min per day 5 times a week experience reduced atrophy in the medial temporal lobe, a key area for memory and executive function [54]. Although the physical activity levels reached in this investigation did not amount to the recommended thresholds presented above, this pilot was concluded after 16 weeks, and further continuation may have allowed participants to continue to reach these levels. Larger-scale investigations are required to better understand the longer-term impact of the Neurotrack MHP on physical activity patterns.

### Nutrition Outcomes

Nutrition has also been discussed as a primary factor in the prevention of cognitive decline [55-57]. On the basis of the MIND diet published by Morris et al [34], we scored self-reported meals throughout the investigation, with participants averaging a final score of 7.6 (SD 1.7) for their nutritional intake. Although a score of 9.6 or higher has been linked to a 53% reduction in the rate of developing AD, those scoring a 7.5 to 9.5 have been associated with a 35% decreased risk of disease development (for a detailed description of the scoring structure, please see the study by Morris et al [34]). This is important given that *perfect eating* is generally unattainable, and flexibility and fluctuation in dietary patterns is expected. In this investigation, females tracked their nutrition significantly more often than males, while also exhibiting significantly higher overall nutritional scores. Given that there is a previously established positive relationship between the act of tracking and improved health outcomes [58], it may be that simply the act of entering one’s food makes individuals more aware of what they are choosing to consume, thus leading to healthier choices and improved outcomes [59,60]. It is also important to note that females are reported to be more interested in seeking out health-related information, pay more attention to potential worldwide pandemics, and are much more attentive as to how goods purchased in everyday life affect their health than men [61].

### Lesson-Related Outcomes

Although participants were able to revisit lessons as many times as they wished, there were only 17 distinct lessons that could be completed. Distinct lessons completed were significantly related to engagement variables (total number of times physical activity was tracked, total number of times nutrition was tracked, total number of times participants meditated, and total minutes exercised). Previous behavior change programs have reported that the completion of weekly lessons is significantly related to increased health outcomes measures such as weight loss [62-64] and reductions in glycosylated hemoglobin [62,63], indicating that continued use of the Neurotrack MHP may lead to additional health benefits in at-risk individuals.

Furthermore, increased lesson completion during behavior change programs has been significantly linked to subsequent improvements in nutrition and physical activity [65]. The relationship between lesson completion and improved outcomes and engagement is further supported when evaluating participants who either completed less than 10 or 10 or more lessons. Significant differences between these groups were observed for the total minutes exercised, total number of times physical activity was tracked, total number of times nutrition was tracked, and total number of times participants mediated. As a result, it is recommended that future iterations of this program aim to increase the adherence to lesson completion, as the action appears to result in subsequent improvements in other parts of the program.

### Limitations

There are a few limitations associated with this study. First, given that this was a pre-post study design and there was no control arm, we were unable to compare the changes in outcomes between individuals using the Neurotrack MHP with the changes in outcomes between individuals who did not use the program. However, previous studies have reported similar results [58,62,64], suggesting that these results may remain when including a designated control, although this cannot be finalized without future investigation. In addition, as this was a pilot study, the sample size was relatively small, and larger samples are needed to make definitive conclusions. Given the demographic used for this investigation, it should also be noted that there is a high ceiling for cognitive health in this population, making it difficult to expect cognitive changes. This is not deemed an issue for this study given the nature of the pilot; however, future studies should prioritize at-risk individuals to further understand effectiveness.

There is also a potential issue related to bias in the results (ie, individuals who engaged in more healthy behaviors were, in turn, more likely to track these behaviors). Future investigations utilizing in-person research designs should work to further confirm these results. Finally, a note needs to be made about localization. This was an American program being delivered to a Japanese population, and although there was an initial layer of localization applied to the program (translation to Japanese, adjustment of wording to be more culturally applicable, etc), there are additional changes that would have made it more directly applicable to a Japanese audience. For example, berries, which are commonly included in the MIND diet, are not easily found within Japanese grocery stores and even when available, they are expensive for regular consumers. A recent version of the MIND diet was published adapting the traditional model to the Japanese market [66], which may increase the levels of tracking or program adherence. It is recommended that future studies should focus on mastering the complexity of deep localization.

### Conclusions

Overall, the various engagement metrics were significantly correlated, and greater engagement was related to improved nutrition scores and increased time exercising. In addition, the relationships between distinct lessons completed and other engagement metrics suggest that there is value in focusing the program on enhancing lesson completion. This notion is further supported by the fact that individuals who completed 10 or more lessons had significantly greater program engagement than individuals who did not. Finally, females demonstrated greater levels of dietary tracking with simultaneous increases in overall nutritional scores, indicating Japanese females may be more likely to engage with behavior change programs than their male counterparts.

## Abbreviations

AD: Alzheimer disease
FINGER: Finnish Geriatric Intervention Study to Prevent Cognitive Impairment and Disability
MAPT: Multidomain Alzheimer's Prevention Trial
MCI: mild cognitive impairment
MIND: Mediterranean-Dietary Approaches to Stop Hypertension Intervention for Neurodegenerative Delay
MHP: Memory Health Program
PHQ-4: Patient Health Questionnaire-4
SCD: subjective cognitive decline
VC Health: Virtual Cognitive Health
VPC: visual paired comparison
